# Supplementary material for: Community health worker and caregiver experiences and perceptions of a multimodal handheld pulse oximeter used in sick child consultations in rural Burundi: A qualitative evaluation
Source: PLOS Glob Public Health. 2025 Jan 13;5(1):e0002399. doi: 10.1371/journal.pgph.0002399 (PMC11729966; doi:10.1371/journal.pgph.0002399)
Supplement: S2 File — (DOC) [file pgph.0002399.s002.doc]

Evaluation of a Non-invasive Pulse Oximeter to Improve Child Wellbeing Outcomes in Burundi

**CHW Acceptance of Non-invasive Pulse Oximeter for Screening of Temperature, Pulse, Respiration Rate, and Oxygen Saturation**

**Focus Group Discussion (FGD) for CHWs**

**Focus Group: Facilitation Details**

Date: __________________ (D/M/Y) Location: _______________________________

Interview Moderator Name: ___________________________ Position/Job Title: ______________________________

**Focus Group: Demographic Details**

FGD#___________ Age Range: Min ______ Max ________ Average ________

Number of FGD Participants:­­­­­­­­­ ______ Location of FGD:___________________________

Participants’ Sex Distribution: Male ______ Female _______

Number of CHWs who have completed secondary school: ________

-----------------------------------------------------------------------------------------------------------------------------------------------------------------------

**Facilitator’s Welcome: Introduction and Instructions to Participants**

**Welcome** and thank you for volunteering to take part in this focus group. You have been asked to participate as your point of view is important. I realize you are busy, and I appreciate your time.

**Introduction:** This focus group discussion is designed to assess your thoughts and feelings about the non-invasive pulse oximeter monitor which measures temperature, pulse, respiration rate, and oxygen saturation.

**Confidentiality:** I would like to assure you that the discussion will be anonymous. You should try to answer and comment as accurately and truthfully as possible. If there are any questions or discussions that you do not wish to answer or participate in, you do not have to do so; however please try to answer and be as involved as possible.

**Ground Rules:**

- The most important rule is that only one person speaks at a time. There may be a temptation to jump in when someone is talking but please wait until they have finished
- There are no right or wrong answers
- You do not have to speak in any particular order
- When you do have something to say, please do so. There are many of you in the group and it is important that I obtain the views of each of you
- You do not have to agree with the views of other people in the group
- Does anyone have any questions? (answers).
- OK, let’s begin

**Warm Up:**

- First, I’d like everyone to introduce themselves. Can you tell us your name?

-----------------------------------------------------------------------------------------------------------------------------------------------------------------------

**Non-invasive Pulse Oximeter**

**CHW Acceptance Evaluation**

1. What is your experience of using pulse oximeters in children?

__________________________________________________________________________________________________________________________________________________________________________________________________

2. How well do you think caregivers liked the device? How well do you think children liked the device? (Including the music and animals)

__________________________________________________________________________________________________________________________________________________________________________________________________

3. What have been the main issues you’ve encountered when using pulse oximeters?

__________________________________________________________________________________________________________________________________________________________________________________________________

_________________________________________________________________________________________________

4. What have been the things you like most about using the pulse oximeters?

__________________________________________________________________________________________________________________________________________________________________________________________________

_________________________________________________________________________________________________

5. Thinking about the probe, we would like to hear your feedback about some aspects of using them:

- Ease of putting and keeping the probe on the child ease of taking a reading, ease of keeping it clean and storage.

__________________________________________________________________________________________________________________________________________________________________________________________________

- Durability

__________________________________________________________________________________________________________________________________________________________________________________________________

- Ease of keeping it clean and storage.

__________________________________________________________________________________________________________________________________________________________________________________________________

6. Thinking about the oximeter, we would like to hear your feedback about some aspects of using them:

- Was the device easy to use? (If they say no, ask what made it difficult hard to use.)

__________________________________________________________________________________________________________________________________________________________________________________________________

- Ease of reading the display

__________________________________________________________________________________________________________________________________________________________________________________________________

- Durability, battery life and charging

__________________________________________________________________________________________________________________________________________________________________________________________________

- Time taken to get a reading.

__________________________________________________________________________________________________________________________________________________________________________________________________

7. What things would make the probe and pulse oximeter easier to use?

_________________________________________________________________________________________________________________________________________________________________________________________________

8. What things would make the probe and pulse oximeter harder to use?

__________________________________________________________________________________________________________________________________________________________________________________________________

9. How does the device compare to the stop watch for measuring respiration rate?

­­­­­­­­­­_________________________________________________________________________________________________

10. What was your experience sharing the device with other CHWs?

__________________________________________________________________________________________________________________________________________________________________________________________________

11. Is sharing a device possible in the geographical area that you serve? Why or why not?

__________________________________________________________________________________________________________________________________________________________________________________________________

**Conclusion**

- Thank you for participating. This has been a very successful discussion
