# Supplementary material for: Community health worker and caregiver experiences and perceptions of a multimodal handheld pulse oximeter used in sick child consultations in rural Burundi: A qualitative evaluation
Source: PLOS Glob Public Health. 2025 Jan 13;5(1):e0002399. doi: 10.1371/journal.pgph.0002399 (PMC11729966; doi:10.1371/journal.pgph.0002399)
Supplement: S3 File — (DOCX) [file pgph.0002399.s003.docx]

**Evaluating the accuracy, acceptability, and feasibility of the Rad-G Pulse Oximeter to improve integrated community case management of childhood illnesses in Burundi**

Focus Group Discussion (FGD) Codebook

Code System

| **Code System** | **Memo** |
| --- | --- |
| **Code** | **Memo** |
| **CHW's experience using Rad-G (Positive/Negative)** | **Process Code (Color, Blue)**  ***Description:*** This code describes a positive/negative emotion in a categorical coding as indicated by how the community health worker felt on using the Rad-G device.   ***Purpose:*** The purpose of this code is to summarize usability/ feasibility of the Rad-G device on CHW's service to children. |
| **Caregivers experienced using the Rad-G (Positive/Negative)** | **Value Code (Color, Green)**  ***Description:*** This code describes a negative/positive emotion on a categorical theme as expressed by the respondents on how the parents/caregivers reacted to the use of pulse oximeter device on their child/children.  ***Purpose:*** The purpose of this code is to summarize responses indicating that the caregiver/parent felt negative, bad, and/or sad or positive, good, and/or happy while the Rad-G device was being used on their child/children. |
| **Children experience on Rad-G (*positive/negative)** | **Value Code (Color, Green)**  ***Description:*** This code describes a positive/negative categorical emotion expressed by the respondent on how the child/children reacted to the use of pulse oximeter device.  ***Purpose:*** The purpose of this code is to understand the usability of the Rad-G device by children. |
| **Barriers CHWs faced in using the device** | **Group Code (Color, Black)**  ***Description:*** This code groups all the challenges the community health worker encountered while using the device. This code will not have coded segments.  ***Purpose:*** This code summarizes feasibility features of Rad-G device. The purpose of this code is to create a grouping for responses indicating issues that caused unsuccessful/delayed measurements of children e.g. (child agitation, device technicalities, inability to interpret results). |
| **Barriers CHWs faced in using the device > Late display of Respiratory rate results** | **Descriptive Code (Color, Violet)**  ***Description:*** This code records responses on challenges the community health worker encountered regarding instances when the Rad-G device took longer to display respiratory rate results.  ***Purpose:*** The purpose of this code is to summarize responses indicating feasibility of using Rad-G device. |
| **Barriers CHWs faced in using the device > Place to charge the device** | **Descriptive Code (Color, Red)**  ***Description:*** It summarizes the challenges the community health worker encountered regarding difficulty in finding a place to recharge the device. e.g. (being far from the health facility).  ***Purpose:*** The purpose of this code is to summarize feasibility of the Rad-G device. |
| **Barriers CHWs faced in using the device > Inability to interpret some results** | **Process Code (Color, Blue)**  ***Description:*** This code describes the challenges the community health worker encountered regarding interpreting the results displayed for diagnosis.  ***Purpose:*** The purpose of this code is to summarize usability of the Rad-G device by CHWs. |
| **Barriers CHWs faced in using the device > Child agitation** | **Descriptive Code (Color, Blue)**  ***Description:*** This code describes the challenges arising from the children crying or having constant movements or agitation during taking measurements.  ***Purpose:*** The purpose of this code is to summarize feasibility/usability features of the Rad-G device. |
| **Things CHWs like most about using the pulse oximeters** | **Group Code (Color, Black)**  ***Description:*** This code groups all the aspects of the Rad-G pulse oximeter that the community health worker regards as attributes. This code will not have coded segments.  ***Purpose:*** This code summarizes feasibility features of Rad-G device on its attributes. e.g (variety of parameters). |
| **Things CHWs like most about using the pulse oximeters > Variety of parameters** | **Value Code (Color, Green)**  ***Description:*** This code describes a positive emotion expressed by the community health workers on the ability of the device to display respiratoty rate, oygen saturation and pulse rate alongside temperature.  ***Purpose:*** The purpose of this code is to understand the acceptabilitility of the Rad-G device by CHWs. |
| **Things CHWs like most about using the pulse oximeters > The device displays quickly the results** | **Descriptive Code (Color, Red)**  ***Description:*** This code describes a positive emotion expressed by the community health workers on how instant the measurements were displayed on the rad-g pulse device.  ***Purpose:*** The purpose of this code is to understand the feasibility of the Rad-G device by CHWs. |
| **Ease of keeping the sensor on a child's finger** | **Descriptive Code (Color, red)**  ***Description:*** This code is used to summarize short phrases that describe the ease of placing the sensor on the childs finger.  ***Purpose:*** The purpose of this code is to assess feasibility of the Rad-G device by assessing the ease of placing the sensor on a childs finger. |
| **Ease of reading display of the device** | **Descriptive Code (Color, red)**  ***Description:*** This code is used to summarize short phrases that describe the ease of reading the display of measurement results by the Rad-G device.  ***Purpose:*** The purpose of this code is to assess feasibility of the Rad-G device by assessing the ease of reading the display of measurement results. |
| **Time taken to get a reading of the results** | **Descriptive Code (Color, red)**  ***Description:*** This code is used to summarize short phrases that describe the duration it took the Rad-G device to display the results after placing the sensor on a child's finger.  ***Purpose:*** The purpose of this code is to assess feasibility of the Rad-G device. |
| **Durability of the Rad-G device (Breaking)** | **Descriptive Code (Color, red)**  ***Description:*** This code is used to summarize short phrases that describe how durable and hard the Rad-G device is.  ***Purpose:*** The purpose of this code is to assess the ruggability of the Rad-G device in adverse environments. |
| **Ease of keeping the device clean** | **Descriptive Code (Color, red)**  ***Description:*** This code is used to summarize short phrases that describe the ease of keeping the device clean.  ***Purpose:*** The purpose of this code is to assess the ease of maintainance of the Rad-G device. |
| **Ease of keeping the device in a safe place** | **Descriptive Code: (Color, red)**  ***Description:*** This code is used to summarize short phrases that describe the ease of keeping the device in a safe place.  ***Purpose:*** The purpose of this code is to assess storability / feasibility of the Rad-G device. |
| **Concerns that the device might get lost/stolen** | **Value Code (Color, Green)**  ***Description:*** This code describes a positive/negative emotion expressed by the community health workers on how secure or unsecure their Rad-G device is from theft or getting lost.  ***Purpose:*** The purpose of this code is to understand the concerns on safeguarding of the Rad-G device by CHWs. |
| **Battery durability** | **Descriptive Code (Color, red)**  ***Description:*** This code is used to summarize short phrases that describe how durable/long-lasting the battery of the Rad-G device is.  ***Purpose:*** The purpose of this code is to assess the reliability of the Rad-G device. |
| **Number of times the battery has been charged** | **Descriptive Code (Color, red)**  ***Description:*** This code is used to summarize short phrases that describe how durable/long-lasting the battery of the Rad-G device is.  ***Purpose:*** The purpose of this code is to assess reliability of the Rad-G device. |
| **Where did you charge the device** | **Descriptive Code (Color, red)**  ***Description:*** This code is used to summarize short phrases that describe how/where the CHWs charged the Rad-G device whenever it was low on battery.  ***Purpose:*** The purpose of this code is to assess feasibility of charging the Rad-G device. |
| **Recommendations to make the probe and device easier to use** | **Concept Code (Color, Violet)**  ***Description:*** This code is used to summarize short phrases that describe the reccommendations that the CHWs suggested on making the probe sand the device easier to use.  ***Purpose:*** The purpose of this code is to draw suggestions on improving acceptability/ usability of the Rad-G device. |
| **Ease of sharing the device with other CHWS** | **Value Code (Color, Green)**  ***Description:*** This code describes how the CHWs perception on the ease of sharing the Rad-G device with other CHWs.  ***Purpose:*** The purpose of this code is to understand the sharing workability/feasibility of the Rad-G device. |
| **Possibility of sharing the device in your geographical area** | **Value Code (Color, Green)**  ***Description:*** This code describes how the CHWs perceive the possibility of sharing the device withing CHWs in the same geographical area could be.  ***Purpose:*** The purpose of this code is to understand the sharing workability/ feasibility of the Rad-G device. |
| **Ability to use the device correctly** | **Concept Code (Color, Yellow)**  ***Description:*** This code describes CHHs self efficacy by evaluating their intellectual ability to use the device correctly.  ***Purpose:*** The purpose of this code is to understand the usability of the Rad-G device on CHWs capacity level. |
| **Self-efficacy: CHWS confidence in interpreting measurements** | **Concept Code (Color, Yellow)**  ***Description:*** This code is used to summarize short phrases that describe how confident the CHWs indicates on correctly interpreting results of measurements on Rad-G device.  ***Purpose:*** The purpose of the code is to assess the usability of the Rad-G device for CHWs service to children by assessing their confidence in the ability to correctly interprate results correctly. |
| **Ease of diagnosing childhood Pneumonia using Rad-G device** | **Value Code (Color, Green)**  ***Description:*** This code describes a emotions and recounts of CHWs on how the Rad-G device has improved the process of diagnosing childhood pneumonia.  ***Purpose:*** The purpose of this code is to understand the usability of the Rad-G device by CHWs. |
| **Rad-G device efficacy in referring children** | **Value Code (Color, Green)**  ***Description:*** This code describes phrases that CHWs recount on how Rad-G made it easy for them to make a decision on refering a child for further medical assistance.  ***Purpose:*** The purpose of this code is to understand the usability/efficacy of the Rad-G device by CHWs. |
| **I do/not recommend using the device for ICCM consultations** | **Value Code (Color, Green)**  ***Description:*** This code describes a positive/negative opinion that a respondent gave regarding the adoption/use of Rad-G in the iCCM consultations.  ***Purpose:*** The purpose of this code is to understand the acceptability of the Rad-G device by the repsondents.children. |
| **Trust on accuracy of the device** | **Emotion code (Color, Turquois)**  ***Definition:*** This code describes a positive/ negative emotion expressed by the respondent.   ***Purpose:*** Apply this code when the caregiver recalls an emotion that is positive, good, and/or happy or negative, bad and/or sad categorically. |
| **Parents perceptions on health workers using Rad-G** | **Emotion code (Color, Turquois)**  ***Definition:*** This code describes a positive/ negative emotion expressed by the parent/caregiver on how they felt about an health provider using a Rad-G device on their child/children.   ***Purpose:*** Apply this code categorically when the parent recalls an emotion that is positive, good, and/or happy or negative, bad and/or sad. |
| **CHW's using the Rad-G device were compassionate** | **Emotion code (Color, Turquois)**  ***Definition:*** This code captures phrases indicating the compassionate nature of CHWs when attending to children to take measuremnts.   ***Purpose:*** This code serves to summarize occassions when health providers applied compassion to enable a smooth measuring process and satisfied caregivers. |
|  |  |
