# Supplementary material for: Community health worker and caregiver experiences and perceptions of a multimodal handheld pulse oximeter used in sick child consultations in rural Burundi: A qualitative evaluation
Source: PLOS Glob Public Health. 2025 Jan 13;5(1):e0002399. doi: 10.1371/journal.pgph.0002399 (PMC11729966; doi:10.1371/journal.pgph.0002399)
Supplement: S1 Checklist — (DOCX) [file pgph.0002399.s004.docx]

Inclusivity in global research

PLOS’ policy on inclusivity in global research aims to improve transparency in the reporting of research performed outside of researchers’ own country or community and ensures that PLOS publications reporting global research adhere to high standards for research ethics and authorship. Authors of relevant research articles may be asked to complete the questionnaire below, which outlines ethical, cultural, and scientific considerations specific to inclusivity in global research. This questionnaire may be requested when researchers have travelled to a different country to conduct research, if research uses samples collected in another country, research with Indigenous populations or their lands, or if research is on cultural artefacts. Researchers travelling to another country solely to use laboratory equipment will not normally be required to complete the questionnaire. However, the questionnaire can be requested at the journal’s discretion for any submission – if you have been requested to complete this questionnaire by the PLOS journal you submitted to, please do so.

Please complete the questionnaire below and include this as a Supporting Information file with your manuscript. Note that if your paper is accepted for publication, this checklist will be published with your article in the supporting information files. Please ensure that you reference the checklist in the main body of your manuscript. We suggest adding a subsection ‘Inclusivity in global research’ to your Methods section and adding the following sentence: “Additional information regarding the ethical, cultural, and scientific considerations specific to inclusivity in global research is included in the Supporting Information (SX Checklist)”

The questions have been designed to be applicable to a wide range of study types, and there are subsections for both human subjects research and non-human subjects research. If any of the questions are not relevant to your research please mark them as “N/A” as appropriate.

**Ethical considerations, permits and authorship**

*This section is applicable to all research types.*

Provide details as to who granted permissions and/or consent for the study to take place in the Methods section of your manuscript. This should include the names of **all** ethics boards, governmental organizations, community leaders or other bodies that provided approval for the study. If individuals provided approval refer to these people by their role or title but do not list their name(s).

Reported on page number 8 and 9 in the manuscript: Ethical clearance for this study was obtained from the Burundi National Ethics Committee for the Protection of Human Beings participating in behavioral research and the statistical visa from the Burundi Institute of Statistics and Economic Studies (ISTEEBU) prior to commencing the project. A pilot committee consisting of nine doctors from the MOH and WV Burundi was established to supervise the study and to determine if the study findings justified efforts to scale the innovation and adapt iCCM protocols. A technical committee consisting of delegates from the Burundi National of Public Health, UNICEF, WHO, and WV Burundi was also established to provide quality assurance to the study design, protocol, instruments, and study findings.

If there were any deviations from the study protocol after approval was obtained please provide details of these changes in the Methods section of your manuscript.
Did this study involve local collaborators that are residents of the country where the research was conducted or members of the community studied? If you do not have any authors from said communities, please provide an explanation for this below.

Yes, the study was implemented by local Burundian staff from World Vision Burundi and UNICEF Burundi. Leocadie Nivyindika (UNICEF Burundi), Vital Habonimana (World Vision Burundi), and Dionis Nizigiyimana (Burundi National Institute of Public Health) have been listed as co-authors. Dr. Nivyindika is listed as second author.

Reported on page number (N/A): There were no deviations from the study protocol.

Everyone listed as an author should meet PLOS’ criteria for authorship and all individuals who meet these criteria should be included in the author byline, rather than the acknowledgements. For further information please see the journal’s Authorship Policy.

**Human subjects research (e.g. health research, medical research, cross-cultural psychology)**

Did you obtain written informed consent from a representative of the local community or region before the research took place? How did you establish who speaks for the community? Details of written informed consent obtained from study participants should be reported separately in the Methods section of your manuscript.

World Vision primary goal is to partner with communities to overcome poverty and injustice. In Burunid, World Vision obtained written consent through a five-year memorandum of understanding (MOU) to partner with the Ministry of Health, including local health facilities within the targeted communities to address poor health and nutrition outcomes, especially among children under five years. The first MOU was signed in 2018 and then renewed for additional five years in Dec. 15^th^, 2023. When speaking to communities, we work with Village Leaders, Religious Leaders, and Caregivers, giving special consideration to elevating the voices of women and children. We take a grassroots approach to designing, implementing, and evaluating our programming. For more information on our community-based approaches in Burundi, please see this [link.](https://www.wvi.org/burundi/about-us) CHWs provided informed written consent to participate in the study, and verbal consent to participate in the FGD discussions, which were documented and witnessed by the FGD Facilitator and FGD Notetaker, the Burundi National Ethics Committee for the Protection of Human Beings.

How did members of the local community provide input on the aims of the research investigation, its methodology, and its anticipated outcome(s)?

Yes, the study was designed in collaboration with World Vision Burundi, WHO Burundi, UNICEF Burundi, and the Burundi Institute of Statistics and Economic Studies, which is critical for the adoption and scaling of promising innovations. World Vision International developed the original study protocol, and then we met several times with Burundian stakeholders for their input and recommendations and refined the study plan accordingly. This paper represents the research co-designed, co-implemented, and co-evaluated by Burundi stakeholders, and follows World Vision International’s guidelines on research equity that seeks to conduct research in such a way that does not perpetuate paternalism, colonialism, racism, and white saviorism. We believe in investing in community-centered research that allows participants to provide their perspectives and elevate their voices, and we strive to link community-driven agendas to appropriate and responsive research. We approach research from a place of humility.

When engaging with the local community, how did you ensure that the informed consent documents and other materials could be understood by local stakeholders?

The consent forms were shared in Kirundi, as Kirundi is more commonly understood than French in the local communities participating in this study. FGD discussions were conducted and recorded in Kirundi and then translated into French and English.

Will the findings of the research be made available in an understandable format to stakeholders in the community where the study was conducted (e.g. via a presentation, summary report, copies of publications, etc.)? Please provide details of how this will be achieved.

Yes, we developed reports and slide decks with our study findings in French to share with Burundi UNICEF, Burundi WHO, the Burundi Institute of Statistics and Economic Studies, and the Burundi Institute of Public Health. All World Vision Burundi staff speak French. We also used data visualizations to ensure that the findings could be more easily understood, and presentations of study findings were conducted in French.

**Non-human subjects research using specimens/ animals collected as part of the study, or those housed in archival collections. Examples include archaeology, paleontology, botany and zoology.**

Did the permission you obtained from a local authority to perform the study include an agreement on access to outputs and benefit sharing? This may include procedures to enable fair distribution of the benefits and resources arising from the research performed. Please include any details of Prior Informed Consent and Benefit Sharing Agreements obtained. These may be required by field-specific regulations, for example the Convention on Biological Diversity (CBD) and the associated Nagoya Protocol.

N/A

If the material used in your study was imported, please A) provide the year it was imported and B) indicate whether permits were obtained to import/export the materials used, C) provide details of any permits obtained. If this information is not available, please indicate this.

N/A

If you used archival specimens, please state how the material used in your study was acquired by the institute it is held in and provide details of any permits obtained for the original excavations/ sample collection. If this information is not available, please indicate this.

N/A

How was the potential cultural significance of the materials collected in your study to local communities considered in your research design? Were Indigenous peoples and/or local researchers and institutions involved with archaeological excavations / collection of specimens? If so, please provide a description of their involvement.

N/A

If your manuscript includes photographs of human remains please indicate whether authors obtained permission from descendants or affiliated cultural communities to do so.

N/A
