# Supplementary material for: Community health worker and caregiver experiences and perceptions of a multimodal handheld pulse oximeter used in sick child consultations in rural Burundi: A qualitative evaluation
Source: PLOS Glob Public Health. 2025 Jan 13;5(1):e0002399. doi: 10.1371/journal.pgph.0002399 (PMC11729966; doi:10.1371/journal.pgph.0002399)
Supplement: Attachment — Submitted filename: Response to Reviewers.docx [file pgph.0002399.s005.docx]

Response to Reviewers

June 20^th^, 2024

Dear Reviewer,

Thanks very much for your review. Please find our responses to your comments below in black font.

Reviewer #1: Thanks for the opportunity to review this important piece. Overall very well written and presented study. A few comments to think about and respond to:

- you had the CHWs use the tool for 8 weeks before the FGDs - was this enough time for them to really understand and use it enough to provide rich and complete data? If you have a rationale for this it would be good to state it. It feels a bit of a short time to me and could almost be a limitation - worth considering and addressing in the article.

Thank you for your comment. Each CHW conducted, on average, conducted 20 to 25 iCCM consultations per week, which is around 160 to 200 iCCM consultants over 8 weeks. We felt this number of consultants was sufficient for the CHWs to assess how well they liked or disliked using the devices (acceptability). However, we agree that piloting the devices for longer than 8 weeks, even 6 to 12 months, would allow us to assess more deeply the devices’ durability, especially battery life. We included this in the sentence in the manuscript: “An additional limitation was the relatively short follow-up period of eight weeks for this pilot. Future studies should evaluate the experiences and perceptions of CHWs and caregivers, as well as device durability and performance, including battery life, over a longer time period, such as 6 to 12 months.”

For the FGDs I couldn’t see if you split them between men and women as sometimes there can be a gender bias presented by mixed groups?

This is a good point! We had 18 female CHWs and 12 male CHWs, so we could have separated the FGDs by gender. We did train our FGD Facilitators to be gender-sensitive and to cultivate a discussion where one or two people don’t dominate the discussion. However, I agree that separating the CHW FGDs could generate deeper findings around barriers and facilitators to using the device specifically related to gender. We will consider this for future studies; it’s a good recommendation. I have added this sentence to the manuscript under the Strengths and Limitations section: “Another limitation is that we did not separate the CHW FGDs by gender. “To mitigate gender bias, FGD facilitators were trained on group dynamics and ground rules to ensure every participant had the opportunity to share in a safe and confidential environment. However, future studies could apply a gender framework when exploring barriers and facilitators to using pulse oximeters during CHW iCCM consultations.” We also added to the Methods Section that the FGD Facilitators were “trained in group dynamics.”
