# Supplementary material for: Community health worker and caregiver experiences and perceptions of a multimodal handheld pulse oximeter used in sick child consultations in rural Burundi: A qualitative evaluation
Source: PLOS Glob Public Health. 2025 Jan 13;5(1):e0002399. doi: 10.1371/journal.pgph.0002399 (PMC11729966; doi:10.1371/journal.pgph.0002399)
Supplement: Attachment — Submitted filename: Response to Reviewer 2.docx [file pgph.0002399.s006.docx]

Response to Reviewer 2

October 22, 2024

Dear Reviewer,

Thanks very much for your review. Please find our responses to your comments below in black font.

**Comments to the Author**

Reviewer #2: The paper investigates the acceptability and feasibility of using a multimodal handheld pulse oximeter by Community Health Workers (CHWs) in rural Burundi during sick child consultations. The study focuses on CHWs’ and caregivers’ experiences using the device to assess children aged 6-59 months as part of Integrated Community Case Management (iCCM). The oximeter measures multiple parameters including pulse rate, oxygen saturation, respiration rate, and temperature, aimed at improving the diagnosis of childhood illnesses, particularly pneumonia. It is a well-written manuscript. However, the current version needs to be revised before it is considered for publication.

Thank you for your thoughtful and excellent questions and comments, as it strengthens our paper. We have responded to each point below and made the necessary changes in the manuscript (using tracked changes).

1. The paper should define more precise research questions or hypotheses upfront. While the study explores various aspects of pulse oximeter use, a clearer articulation of objectives will help frame the findings more effectively.

Thank you, we agree. We created a new sub-heading, 2.1. entitled “Research Objectives” and added the research question, “What are the barriers (obstacles) and facilitators (opportunities) to utilizing pulse oximetry during sick child visits conducted by community health workers in low-resource settings in Burundi?” We’ve also stated in the manuscript that we “conducted a prospective pilot study and used qualitative research to explore the acceptability and feasibility of a non-invasive pulse oximeter used during CHW iCCM consultations.”

2. Kindly expand on the rationale for choosing the sample size of 32 CHWs and clarify whether this size is sufficient for saturation in qualitative analysis. Including more information on how sampling choices may have influenced the findings would be beneficial.

To determine the sample size, we’ve referred to the paper “How Many Focus Groups are Enough? Building an Evidence Base for Nonprobability Sample Sizes”, which can be found [here](https://protect.checkpoint.com/v2/___https://journals.sagepub.com/doi/abs/10.1177/1525822X16639015?journalCode=fmxd___.YzJ1OndvcmxkdmlzaW9uaW5jOmM6bzo4YTYzOGNjODg1ZTgyNjdmM2ZlNDUzMjgzZWE0ZDJiZTo2OjE3ZTg6MDRhMzJlZTQ1MjBkNjlmYzQyMWU3NzBkMTFkMjRhNzU5YjlmNTYzODYwNmVhMjBmZjQzZDNhMjE2NzU3MGM5ZDpwOlQ6Tg). In that paper, the authors state, “ Our analyses revealed that more than 80% of all themes were discoverable within two to three focus groups, and 90% were discoverable within three to six focus groups. Three focus groups were also enough to identify all of the most prevalent themes within the data set.” We used the evidence generated from this paper to justify four FGDs (8 CHWs/FGD) as sufficient to reach saturation. We’ve included this justification (and referenced the paper) in the revised manuscript.

3. The paper notes a particular issue with younger children (6-12 months) but lacks explanation as to why this group was especially problematic. More in-depth analysis of age-specific challenges could help strengthen the discussion.

We stated in the paper, “Child agitation (usually described as crying) was greater among children under one year and a barrier to utilizing the device easily and correctly.” We’ve also added the sentence (with reference), “Infants under one year are more prone to agitation than older age groups due to their limited ability to self-regulate emotions, as their neurobiological systems and coping mechanisms are still developing, making them more sensitive to stress and stimuli.” We also added a study (with reference) in Ethiopia that reported a similar finding as our study: “Similar findings around child calming difficulties among health extension workers were reported in a cross-sectional study evaluating usability and acceptability of a multimodal respiratory rate and a pulse oximeter device in case management of children with symptoms of pneumonia in Ethiopia.”

4. The paper makes comparisons with other studies (e.g., on respiration rate accuracy in other countries). More direct comparisons between the study’s findings and those in similar contexts could enrich the analysis and provide a broader perspective.

Thank you. we have added a direct comparison between our study’s findings and findings from other studies in similar contexts around child agitation. I added this sentence (with reference) in the Discussion Section, “Our findings are similar to a study conducted in Malawi and Bangladesh, which found that movement among physically smaller children caused pulse oximetry measurement challenges.” We also added another study (with reference) in Ethiopia, which assessed the usability and acceptability of a multimodal respiratory rate and pulse oximeter device in case management of children with symptoms of pneumonia, as this study found similar child calming difficulties. From our best knowledge from the Burundi MOH and UNICEF Burundi, we are the first study to explore pulse oximetry during CHW sick child visits in Burundi.

Under the Strengths and Limitations section, we also added this sentence (with reference), “Although designing the device prototype was outside the scope of this study, utilizing human-centred design (HCD) approaches, like the study in South Africa that used HCD to design a smartphone-based pulse oximeter for children (32), can be valuable method to facilitate pragmatic and tangible design changes.”

5. While the paper mentions that pulse oximetry may be more feasible at the health facility level, this argument could be expanded. A more detailed exploration of the challenges to scaling the use of oximeters at the community level, as well as possible solutions, would add depth to the discussion.

We agree. In conclusion, we’ve added, “Future studies should evaluate challenges to bringing pulse oximetry to scale, including the device durability and performance over a longer time period. Finally, adopting pulse oximetry in sick child consultations within Burundi can improve the quality of care by enabling early detection of hypoxemia, allowing for timely interventions and better management of severe respiratory conditions.” For challenges around scaling and possible solutions, we’ve stated this in the paper, “Other recommendations included scaling pulse oximeters to health centers, device maintenance plans, solutions for keeping probes clean, a raincoat to shield the device from rain, having a user interface in the local language (Kirundi), and access to a light source (flashlight) to be able to use the device at night.

6. The paper would benefit from a more thorough discussion of its limitations. For example, limitations in terms of sample size, generalizability to other regions, or potential biases introduced by the qualitative methodology should be explicitly addressed.

Thank you; this was also a suggestion by the first Reviewer (around gender bias). We’ve added limitations on gender bias and social-desirability bias. We’ve also included limitations around generalizability, a relatively short follow-up period, and limiting the study to participants 6 to 59 months. The section on limitations now states:

“A limitation of this study is that it was potentially subject to social-desirability bias, with caregivers and CHWs expressing opinions that they thought the FGD facilitators wanted to hear. To minimize this potential limitation, the purpose of the study was explained to the participants during the consent process, and they were encouraged to share their points of view as truthfully as possible. Another limitation is that we did not separate the CHW FGDs by gender. To mitigate gender bias, FGD facilitators were trained on group dynamics and ground rules to ensure every participant had the opportunity to share in a safe and confidential environment. However, future studies could apply a gender framework when exploring barriers and facilitators to using pulse oximeters during CHW iCCM consultations. An additional limitation was the relatively short follow-up period of eight weeks (from receipt of devices to FGDs) for this pilot. Future studies should evaluate the experiences and perceptions of CHWs and caregivers, as well as device durability and performance, including battery life, over a longer time period, such as 6 to 12 months. However, initial user experiences can impact longer-term adoption and uptake, and our findings provide important insights relevant to implementation considerations. This study was also limited to sick child encounters among 6-59 month-olds, and additional studies could examine the utility of a multimodal oximeter among children <6 months of age as this age group tends to experience higher rates of severe illness and mortality. Additionally, our study was conducted in a very rural and mountainous part of the country, and our findings may not be generalizable to other settings.”

7. There are issues with the referencing (e.g., inaccurate references regarding pneumonia burden). Ensuring all references are accurate and align with the claims made in the paper will increase its credibility.

Thank you for catching this mistake! We have changed the manuscript to: “In Burundi, the 5^th^ lowest country on the Human Development Index (2), malaria, respiratory disease (including pneumonia), and acute diarrhea account for an estimated 93% of all hospitalizations of children ages 1 to 59 months.” We had mistakenly written “2 to 59 months”, but the correct age group is “1 to 59 months.” Also, the paper’s authors referred to pneumonia as “lung disease,” which encompasses pneumonia. We’ve revised the statement to reflect the paper’s description of the illnesses. We’ve also checked the other references to ensure they are correct. We found that reference 11 was missing and reference 12 was missing the URL. We have corrected those mistakes.

8. The conclusion should more clearly synthesize the implications of the findings. A stronger link between the findings, recommendations for policy/practice, and suggestions for future research would provide a more impactful ending.

Thank you; in the paper, we’ve stated, “However, more research is needed to determine community-level and health facility-level child morbidity and mortality, care-seeking practices, and where the greatest burden of severe illness is encountered to inform where diagnostic tools such as multifunction oximeters can be best utilized in low-resource settings.” we’ve also added, “Future studies, utilizing HCD approaches, should evaluate challenges to bringing pulse oximetry to scale, including the device durability and performance over a longer time period.” For policy and programmatic implications, we’ve concluded with, “Finally, adopting pulse oximetry in sick child consultations within Burundi can improve the quality of care by enabling early detection of hypoxemia, allowing for timely interventions and better management of severe respiratory conditions.”
